# Cost-benefit analysis of aquaculture breeding programs

**DOI:** 10.1186/s12711-018-0372-3

**Published:** 2018-01-29

**Authors:** Kasper Janssen, Helmut Saatkamp, Hans Komen

**Affiliations:** 1 Animal Breeding and Genomics, Wageningen University and Research, Droevendaalsesteeg 1, 6708 PB Wageningen, The Netherlands; 2 Business Economics Group, Wageningen University and Research, Hollandseweg 1, 6706 KN Wageningen, The Netherlands

## Abstract

**Background:**

Profitability of breeding programs is a key determinant in the adoption of selective breeding, and can be evaluated using cost-benefit analysis. There are many options to design breeding programs, with or without a multiplier tier. Our objectives were to evaluate different breeding program designs for aquaculture and to optimize the number of selection candidates for these programs.

**Methods:**

The baseline was based on an existing breeding program for gilthead seabream, where improvement of the nucleus had priority over improvement of the multiplier tier, which was partly replaced once every 3 years. Alternative breeding programs considered were annual multiplier tier replacement, annual multiplier tier replacement with priority on improvement of the multiplier tier, and a program without a multiplier tier. Cost-benefit analyses were performed to compare breeding programs. The outcomes were used to describe relationships between profitability and the number of selection candidates, length of the time horizon, and production output, and to estimate the optimum numbers of selection candidates.

**Results:**

The baseline breeding program was profitable after 5 years and reached a net present value of 2.9 million euro in year 10. All alternative programs were more profitable up to year 17. The program without a multiplier tier was the most profitable one up to year 22, followed by the program with annual multiplier tier replacement and nucleus priority. The optimum number of selection candidates increased with the length of the time horizon and production output.

**Conclusions:**

The baseline breeding program was profitable after 5 years. For a short time horizon, putting priority on improvement of the multiplier tier over the nucleus is more profitable than putting priority on nucleus improvement, and vice versa for a long time horizon. Use of a multiplier tier increases the delay between costs made for selection and resulting benefits. Thus, avoiding the use of a multiplier tier will increase the profitability of the breeding program in the short term. The optimum number of selection candidates increases with the length of the time horizon and production output. Using too many selection candidates relative to the optimum leads to less reduction in profitability than using too few selection candidates.

## Background

In European aquaculture, most breeding programs are operated by private companies, i.e. the governments’ involvement is limited [1, 2]. Profitability of breeding programs, therefore, is a key determinant in the adoption of selective breeding [3]. Cost-benefit analysis can be used to evaluate the effectiveness of an investment, to find its optimal scale, and to identify its constraints [4]. The theory for cost-benefit analysis of breeding programs, in particular for livestock species, was developed by Hill [5], Moav [6], Weller [7], and Wilton et al. [8]. The general concept is that benefits and costs of a breeding program are expressed relative to a baseline scenario without genetic improvement. Costs include investments for husbandry and testing facilities, rearing of selection candidates, trait recording, and genetic analysis. Benefits follow from the increase in genetic levels of traits, the economic values of these traits, and production output of the company, market, or industry. Benefits are permanent and cumulative, but delayed relative to the costs incurred to implement selection. To account for differences in timing of benefits and costs, these are discounted to their present values. The difference in present values of benefits and costs—the net present value—for a given time horizon is a measure of the profitability of the breeding program.

Genetic improvement increases farm profit, either via cost reduction per unit product, increased production output, or a combination of both. When breeding is a highly specialized and concentrated activity, such as in salmonids and livestock [2, 9], genetic progress is not necessarily reflected in the market price of eggs, young animals, or parent stock [10]. Instead, the benefits of the breeding program are distributed between the breeding company and its clients, such that the minimum proportion of benefits accrued by the breeding company covers its costs. Integrated companies accrue all benefits from genetic progress generated by the breeding program.

Genetic progress can be disseminated with or without a multiplier tier. Generally, pig and poultry breeding programs consist of a nucleus and one or more multiplier tiers. The nucleus consists of various pure lines that are differentially selected. For example, a sire line may be selected for lean tissue growth and a dam line for reproduction. In the multiplier tier, crossbreeding is performed to exploit heterosis, and market-specific crosses are made to meet the needs of different markets. Because of the limited reproductive ability of pigs and poultry, multiplier tiers are required to disseminate genetic progress [11, 12]. In aquaculture, breeding programs with and without a multiplier tier exist. Specialized breeding companies for salmonids usually make use of a multiplier tier, partly because the fecundity of salmonids is insufficient to supply the entire market directly from the nucleus. Integrated breeding companies control the entire process from reproduction to harvest and operate a breeding program as an integrated part of the process [2]. Some integrated companies use a multiplier tier, while others do not. When a multiplier tier is used, the highest ranking animals may be used for nucleus replacement and the next tier for multiplier tier replacement, or vice versa. It is unclear which strategy is most profitable. Integrated companies that do not use a multiplier tier use the nucleus to supply production. A multiplier tier can result in delay between genetic progress and its dissemination [13] and may thereby negatively affect profitability of a breeding program. Thus, studying the economic consequences of implementing a multiplier tier is relevant in aquaculture breeding programs.

For integrated companies, the general objective of investing in a breeding program is maximization of the net present value. Benefits are proportional to the selection intensity and production output of the company, while costs are largely proportional to the number of selection candidates. Therefore, there is an optimum number of selection candidates that maximizes net present value.

The first objective of this study was to evaluate the effectiveness of investing in a breeding program by an integrated aquaculture company. The second objective was to evaluate the profitability of alternative breeding program designs. The third objective was to describe the relationship between net present value and the number of selection candidates, length of the time horizon, and production output, and to estimate the optimum number of selection candidates.

## Methods

The baseline for the analyses was based on an existing breeding program for gilthead seabream. Improvement of the nucleus had priority over the multiplier tier, which was partly replaced once every three years. Alternative breeding programs were annual multiplier tier replacement, annual multiplier tier replacement with priority on improvement of the multiplier tier, and a breeding program without a multiplier tier. In all breeding programs, the number of parents per selection round and the number of selection candidates were equal to those in the baseline program, hence the selection intensity and selection index remained the same over breeding programs.

### Structure of the baseline breeding program

**Fig. 1:**
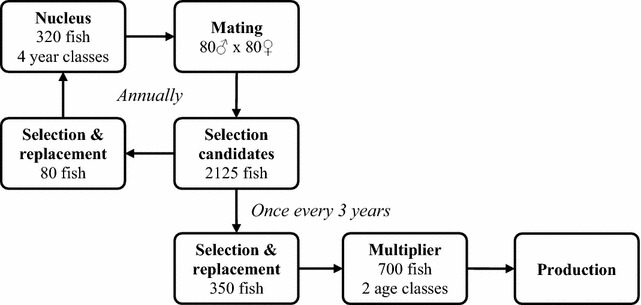
Schematic overview of the baseline breeding program

The baseline breeding program was based on the existing breeding program of the integrated company Andromeda S.A., one of the largest producers of gilthead seabream. A schematic overview of this breeding program is in Fig. 1. It consists of a nucleus of 320 fish, comprised of four year classes, with an overall male to female ratio of 1:1. Because seabream is a protandrous hermaphrodite, younger year classes consist primarily of males and older year classes primarily of females. Thus, broodstock may initially contribute to offspring as males and later as females, which explains the relatively large size of the nucleus. Every year, 80 males and 80 females are selected from the nucleus and distributed over eight spawning tanks according to a mating design that manages contributions of parents. Seabream is a batch spawning species that can produce 20,000 to 80,000 eggs per day for a period of up to three months [14]. Equal quantities of fertilized eggs from all spawning tanks are collected on the same day, pooled, and larvae are reared communally. After weaning, 2500 juveniles are selected at random, tagged, fin clipped, and transferred to a sea cage. Microsatellite analyses are used for pedigree reconstruction. Selection is performed after 18 months when the fish reach 400 g. Every selection round, 2215 selection candidates survive of which the 80 highest ranking fish based on aggregate genotype are selected as replacements for the oldest year class in the nucleus. Once every three years, an additional 350 fish of the next tier are selected to replace half of the multiplier tier, which is used to produce juveniles for production.

### Genetic selection differential

The genetic selection differential, i.e. the difference in the mean value of the aggregate genotype of selected individuals relative to the mean of all selection candidates, was first predicted from the sum of products of genetic gain per trait per selection round and economic values. The breeding goal included three traits: thermal growth coefficient (TGC), thermal feed intake coefficient (TFC), and mortality rate (M) [15]. TGC is a measure of growth rate corrected for initial bodyweight and the sum of lifetime daily temperatures. TFC is a measure of feed intake rate corrected for initial bodyweight and the sum of lifetime daily temperatures. M is mortality rate in percent per day. Baseline trait levels, genetic parameters, and economic values are in Table 1. Estimation of genetic parameters for TGC and TFC is described in “Appendix 1”. Genetic and phenotypic correlations between TGC and TFC were assumed to be equal to 0.8 and 0.9, respectively (“Appendix 1”). M was assumed to be uncorrelated to TGC and TFC.

**Table 1:** Traits, trait levels, genetic parameters, and economic values of traits used in the breeding program

Trait	Baseline trait level (trait unit)	Phenotypic variance (trait unit^2^)	Heritability	Economic value (€ (trait unit)^−1^ (ton production)^−1^) [15]
TGC	12.6	2.64	0.34	400
TFC	8.25	1.17	0.26	− 450
M	0.030		0.17^a^	− 7700

TGC = Thermal growth coefficient (g^2/3^/(day degrees·1000))

TFC = Thermal feed intake coefficient (g^0.544^/(day degrees·1000))

M = Mortality rate (%/day)

^a^Liability scale [16]

Genetic gains in TGC and TFC in the nucleus were predicted using SelAction [17], applying 1-stage truncation selection on estimated breeding values. Common environmental effects were set to zero. Genetic gain was predicted as if 80 males and 81 females produce offspring (SelAction does not allow half-sibs groups with equal numbers of males and females). The number of surviving selection candidates was 2125. The numbers of full-sib and half-sib records per selection candidate were estimated from stochastic simulation. With eight spawning tanks each holding 10 males and 10 females, there were 800 possible combinations of parental pairs. A total of 2125 offspring were simulated with each parental pair having an equal chance to contribute offspring. Over 1000 simulations, per selection candidate the mean number of full-sibs was 2.7 and the mean number of half-sibs was 50. The average number of dams of half-sibs was 8.4. In SelAction, a full-sib group of 3 and a half-sib group of 50 originating from eight dams were thus included. Although in reality younger year classes consist primarily of males and older year classes primarily of females, here an abrupt sex change from all male to all female was assumed at the age of 4 years. Thus, selected proportions (*p*) were set to 0.0376 for both sexes. The selection index contained records of TGC based on own performance, best linear unbiased predictions (BLUP) of breeding values of the parents, and records of TGC on three full-sibs and 50 half-sibs. No phenotypes for TFC were recorded. Genetic gain in M was predicted based on mass selection on cumulative mortality, where the selected proportion equalled the surviving fraction of selection candidates and genetic correlations with other traits were assumed to be zero (“Appendix 2”).

The genetic selection differential as predicted here account neither for non-linear relationships between trait levels and change in farm profit nor for interactions between traits. To account for these, the genetic selection differential was also predicted using the bio-economic model described in Janssen et al. [15] by using baseline trait levels and trait levels after one round of selection as inputs. The genetic selection differential was calculated as the increase in gross margin between the baseline and after one round of selection, divided by the volume of fish production before genetic improvement.

### Gene flow

Gene flow [18, 19] was used to simulate increases in the genetic level of each age class of the nucleus and multiplier tier over time. In the nucleus, the 5-year old female year class was replaced every year by 80 2-year old selected males. All year classes in the nucleus were assumed to have equal contributions to selected males, hence mortality in year classes was ignored. A vector ${\mathbf{n}}_{\left( t \right)}$ was defined to simulate the flow of genes between age classes over time. The elements in ${\mathbf{n}}_{\left( t \right)}$ defined the genetic level in each year class of the nucleus at time $t$ (years). The length of ${\mathbf{n}}_{\left( t \right)}$ was five: one for 1-year old selection candidates and four for the 4-year classes.

We defined a matrix ${\mathbf{P}}$ that described the flow of genes due to reproduction and aging, and a vector ${\mathbf{s}}$ that described the increase in breeding values due to selection, such that:

1 $${\mathbf{n}}_{\left( t \right)} = {\mathbf{P}} \cdot {\mathbf{n}}_{{\left( {t - 1} \right)}} + {\mathbf{s}},$$

2 $${\mathbf{P}} = \left[ {\begin{array}{*{20}c} 0 \\ 1 \\ 0 \\ 0 \\ 0 \\ \end{array} \begin{array}{*{20}c} {0.25} \\ 0 \\ 1 \\ 0 \\ 0 \\ \end{array} \begin{array}{*{20}c} {0.25} \\ 0 \\ 0 \\ 1 \\ 0 \\ \end{array} \begin{array}{*{20}c} {0.25} \\ 0 \\ 0 \\ 0 \\ 1 \\ \end{array} \begin{array}{*{20}c} {0.25} \\ 0 \\ 0 \\ 0 \\ 0 \\ \end{array} } \right],$$

3 $${\text{and}}\,{\mathbf{s}}^{{\prime }} = \left[ {\begin{array}{*{20}l} S \hfill & 0 \hfill & 0 \hfill & 0 \hfill & 0 \hfill \\ \end{array} } \right].$$

where $${\mathbf{n}}_{\left( 0 \right)}^{{\prime }} = \left[ {\begin{array}{*{20}l} 0 \hfill & 0 \hfill & 0 \hfill & 0 \hfill & 0 \hfill \\ \end{array} } \right]$$, element $P_{ij}$ of matrix **P** is the proportion of genes in age class $i$ at time $t$ that came from age class $j$ at time $t - 1$, and element $S$ of vector ${\mathbf{s}}$ denotes the genetic selection differential.

The multiplier tier was treated as two age classes with a 1:1 sex ratio, originating from two different selection rounds. Both age classes were assumed to contribute equally to juvenile production, hence the genetic level of fish in production facilities was determined by the average genetic level in the multiplier tier. The genetic selection differential for the 350 fish selected for multiplier tier replacement was lower than that for the 80 fish selected for nucleus replacement. For the nucleus, $p = \frac{80}{2125} = 0.0376$, hence selection intensity ($i$) was 2.18. For the $80 + 350 = 430$ fish used for replacement of both the nucleus and multiplier tier, $p = \frac{430}{2125} = 0.202$, hence $i$ was 1.39. For the 350 fish selected for the multiplier tier, $i$ was thus $\frac{430 \cdot 1.39 - 80 \cdot 2.18}{350} = 1.21$. Genetic gains in TGC and TFC were proportional to $i$, but genetic gain in M was not because $i$ for M depended on the surviving proportion. Thus, the genetic selection differential $S$ was split between the genetic selection differential due to improvement of TGC and TFC ($S_{{{\text{TGC}}, {\text{TFC}}}}$) and the selection differential due to improvement of M ($S_{\text{M}}$). The genetic selection differential in the multiplier tier was expressed relative to $S_{{{\text{TGC}}, {\text{TFC}}}}$ and $S_{\text{M}}$ in the nucleus. A vector ${\mathbf{m}}_{\left( t \right)}$ was defined to simulate the flow of genes towards and within the multiplier tier. The elements in ${\mathbf{m}}_{\left( t \right)}$ defined the genetic level in each age class at time *t* (year). The length of ${\mathbf{m}}_{\left( t \right)}$ was two: the two age classes of the multiplier tier. For years without replacement (*t* = 1, 3, 4, 6, 7, etc.), average breeding values did not change, hence:

4 $${\mathbf{m}}_{\left( t \right)} = {\mathbf{m}}_{{\left( {t - 1} \right)}} .$$

The oldest age class in the multiplier tier was replaced at *t* = 2, 5, 8, 11, etc. For these years, we defined a matrix ${\mathbf{R}}$ that described the flow of genes from the nucleus to the multiplier tier, a matrix ${\mathbf{Q}}$ that described the flow of genes in age classes of the multiplier tier due to aging, and a vector ${\mathbf{r}}$ that described the increase in average breeding values due to selection, such that:

5 $${\mathbf{m}}_{\left( t \right)} = {\mathbf{R}} \cdot {\mathbf{n}}_{{\left( {t - 2} \right)}} + {\mathbf{Q}} \cdot {\mathbf{m}}_{{\left( {t - 3} \right)}} + {\mathbf{r}},$$

6 $${\mathbf{R}} = \left[ {\begin{array}{*{20}c} 0 \\ 0 \\ \end{array} \begin{array}{*{20}c} {0.25} \\ 0 \\ \end{array} \begin{array}{*{20}c} {0.25} \\ 0 \\ \end{array} \begin{array}{*{20}c} {0.25} \\ 0 \\ \end{array} \begin{array}{*{20}c} {0.25} \\ 0 \\ \end{array} } \right],$$

7 $${\mathbf{Q}} = \left[ {\begin{array}{*{20}c} 0 \\ 1 \\ \end{array} \begin{array}{*{20}c} 0 \\ 0 \\ \end{array} } \right],$$

8 $${\text{and}}\,{\mathbf{r}}^{{\prime }} = \left[ {\frac{1.21}{2.18} \cdot S_{{{\text{TGC}},{\text{TFC}}}} + S_{\text{M}} \; 0} \right],$$

where $${\mathbf{m}}_{\left( 0 \right)}^{{\prime }} = \left[ {\begin{array}{*{20}l} 0 \hfill & 0 \end{array} } \right]$$, element $R_{ij}$ of matrix **R** is the proportion of genes in each age class $i$ of ${\mathbf{m}}$ at time $t$ that came from age class $j$ of ${\mathbf{n}}$ at time *t* − 2.

### Breeding program with annual multiplier tier replacement

In this breeding program, part of the multiplier tier was replaced annually. Vector ${\mathbf{n}}_{\left( t \right)}$ was defined as before (Eq. 1). The multiplier tier was made up of 4 year classes of 175 fish, hence the length of vector ${\mathbf{m}}_{\left( t \right)}$ was four. For the $80 + 175 = 255$ fish used for replacement of both the nucleus and multiplier tier, $p = \frac{255}{2125} = 0.120$ and $i$ =1.67. For the 175 fish selected for the multiplier tier, $i$ was thus $\frac{255 \cdot 1.67 - 80 \cdot 2.18}{175} = 1.43$. The genetic selection differential was expressed relative to $S_{{{\text{TGC}}, {\text{TFC}}}}$ and $S_{\text{M}}$ in the nucleus in the baseline. Vectors and matrices ${\mathbf{m}}_{\left( t \right)}$, ${\mathbf{R}}$, ${\mathbf{Q}}$, and ${\mathbf{r}}$ were redefined as:

9 $${\mathbf{m}}_{\left( t \right)} = {\mathbf{R}} \cdot {\mathbf{n}}_{{\left( {t - 2} \right)}} + {\mathbf{Q}} \cdot {\mathbf{m}}_{{\left( {t - 1} \right)}} + {\mathbf{r}},$$

10 $${\mathbf{R}} = \left[ {\begin{array}{*{20}c} 0 \\ 0 \\ 0 \\ 0 \\ \end{array} \begin{array}{*{20}c} {0.25} \\ 0 \\ 0 \\ 0 \\ \end{array} \begin{array}{*{20}c} {0.25} \\ 0 \\ 0 \\ 0 \\ \end{array} \begin{array}{*{20}c} {0.25} \\ 0 \\ 0 \\ 0 \\ \end{array} \begin{array}{*{20}c} {0.25} \\ 0 \\ 0 \\ 0 \\ \end{array} } \right],$$

11 $${\mathbf{Q}} = \left[ {\begin{array}{*{20}c} 0 \\ 1 \\ 0 \\ 0 \\ \end{array} \begin{array}{*{20}c} 0 \\ 0 \\ 1 \\ 0 \\ \end{array} \begin{array}{*{20}c} 0 \\ 0 \\ 0 \\ 1 \\ \end{array} \begin{array}{*{20}c} 0 \\ 0 \\ 0 \\ 0 \\ \end{array} } \right],\,{\text{and}}$$

12 $${\mathbf{r}}^{{\prime }} = \left[ {\frac{1.43}{2.18} \cdot S_{TGC,TFC} + S_{M} \;0\; 0\; 0} \right].$$

where $${\mathbf{m}}_{\left( 0 \right)}^{{\prime }} = {\mathbf{m}}_{\left( 1 \right)}^{{\prime }} = \left[ {\begin{array}{*{20}l} 0 \hfill & 0 \hfill & 0 \hfill & 0 \hfill \\ \end{array} } \right]$$.

### Breeding program with priority on improvement of the multiplier tier

In this breeding program, part of the multiplier tier was replaced annually but priority was put on improvement of the multiplier tier over the nucleus. For the multiplier tier, $p = \frac{175}{2125} = 0.082$, $i$ =1.85. For the nucleus, $i = \frac{255 \cdot 1.67 - 175 \cdot 1.85}{80} = 1.28$. Genetic selection differentials were expressed relative to $S_{{{\text{TGC}}, {\text{TFC}}}}$ and $S_{\text{M}}$ in the nucleus in the baseline. Vector ${\mathbf{n}}_{\left( t \right)}$ was defined as in Eq. 1, where vector ${\mathbf{s}}$ was defined as:

13 $${\mathbf{s}}' = \left[ {\frac{1.85}{2.18} \cdot S_{{{\text{TGC}},{\text{TFC}}}} + S_{\text{M}} \; 0\; 0\; 0\; 0} \right].$$

Vector ${\mathbf{m}}_{\left( t \right)}$ was defined as in Eq. 9, where vector ${\mathbf{r}}$ was defined as:

14 $${\mathbf{r}}^{{\prime }} = \left[ {\frac{1.28}{2.18} \cdot S_{{{\text{TGC}},{\text{TFC}}}} + S_{\text{M}} \;0\;0\;0} \right].$$

### Breeding program without a multiplier tier

This was a breeding program without a multiplier tier. We assumed that in the baseline breeding program the size of the nucleus was too small to supply production year round and expansion of the nucleus would relieve this constraint. Thus, the nucleus was expanded to 700 fish, consisting of 4 year classes of 175 fish each. Thus, $p = \frac{175}{2125} = 0.082$ and $i$ =1.85. The number of parents per selection round was kept at 160, hence some fish would not be used to produce selection candidates but only to supply production. The genetic selection differential was expressed relative to $S_{{{\text{TGC}},{\text{TFC}}}}$ and $S_{\text{M}}$ in the nucleus in the baseline. Vector ${\mathbf{n}}_{\left( t \right)}$ was defined as in Eq. 1, where vector ${\mathbf{s}}$ was defined as:

15 $${\mathbf{s}}^{{\prime }} = \left[ {\frac{1.85}{2.18} \cdot S_{{{\text{TGC}},{\text{TFC}}}} + S_{\text{M}} \;0\;0\;0\;0} \right].$$

### Benefits and costs

For breeding programs with a multiplier tier, benefits in year $t$ were calculated as the average of vector ${\mathbf{m}}_{{\left( {t - 1} \right)}}$ multiplied by 5000 tons per year. For the breeding program without a multiplier tier, benefits in year $t$ were calculated as the average of the last four elements of vector ${\mathbf{n}}_{{\left( {t - 1} \right)}}$ multiplied by 5000 tons per year. For all breeding programs, annual benefits were delayed by one extra year, which is approximately halfway between the total duration of larval rearing and grow out.

Instead of considering initial investment and operational expenses separately, as in conventional cost-benefit analysis e.g. [5], all costs were converted to annual costs. By using annual costs, irregular investment costs were smoothed over time and the issue of financing is circumvented. The investment pattern and the way of financing are highly specific to individual companies, hence using annual costs instead of cash-flows improves general applicability of the analyses. Costs were estimated from bookkeeping records of Andromeda S.A. Only costs specifically required for the breeding program were included, hence costs for reproduction that would also be necessary without a breeding program were excluded. For investments in buildings and tanks, an annuity was calculated based on a lifetime of 20 years and an interest rate of 4.5% [20]. Buildings and tanks had salvage values of zero. Costs for husbandry of the nucleus included feed, daily care, and management of the breeding program. Costs for rearing selection candidates included separate rearing, tagging, handling, and quarantine of selected fish. Costs of separate rearing were calculated as the opportunity costs of selling selection candidates minus revenues from selling unselected fish at a discount. Costs of trait recording included all activities required to measure bodyweight. Costs of external services included genetic analysis with microsatellites for parentage assignment and consultancy. For the breeding program without a multiplier tier, annual costs were reduced due to savings on broodstock facilities and husbandry costs. Costs were on average incurred halfway through the 2-year period required for rearing selection candidates, hence costs were incurred for the first time one year after initiation of a breeding program and every year thereafter.

### Cost-benefit analyses

First, cost-benefit analyses were based on annual benefits and costs as of the start of the breeding program. The net present value was calculated as [21]:

16 $$NPV\left( t \right) = \mathop \sum \limits_{j = 0}^{t} \left( {\left( {B_{j} - C} \right) \cdot r^{j} } \right),$$

where $B_{j}$ are benefits in year $j$, $C$ are annual costs, which are constant over time, and $r$ is a discount factor calculated as $r = \frac{1}{1 + d}$ where $d$ is the discount rate. Thus, $NPV\left( t \right)$ gives the sum of all discounted benefits minus the sum of all discounted costs as of the start of a breeding program up to a time horizon of $t$ years. The discount rate was set equal to a risk-free rate of return on private investment of 4.5% per year plus a risk premium of 2% per year, summing to 6.5% per year [22]. The $NPV\left( t \right)$ was calculated up to a time horizon of 20 years.

Second, with the aim of explaining differences between breeding programs and for their later optimization, $NPV$ was approximated algebraically as [based on 5]:

17 $$NPV_{appr} \left( t \right) = \bar{B}\left( {\frac{{r \cdot \left( {r^{y} - r^{t} } \right)}}{{\left( {1 - r} \right)^{2} }} - \frac{{\left( {t - y} \right) \cdot r^{t + 1} }}{1 - r}} \right) - C \cdot \frac{{r - r^{t + 1} }}{1 - r},$$

where $\bar{B}$ is the average increase in benefits per year and $y$ is the average delay between costs made for a selection round and resulting benefits. However, when the benefits of a selection round were incurred exactly was not self-evident. For breeding programs with a multiplier tier, part of the benefits followed from the genetic selection differential in the multiplier tier and another part followed from the genetic selection differential in the nucleus, which was gradually transmitted to the multiplier tier in successive years. Furthermore, in the baseline breeding program, benefits increased only once every three years, instead of annually. To find $y$, Eq. 17 was solved for $NPV_{appr} \left( {100} \right)$, estimated by simulating the increase in genetic level to ${\mathbf{n}}_{{\left( {99} \right)}}$ and ${\mathbf{m}}_{{\left( {99} \right)}}$ using the gene flow model described above.

### Optimum number of selection candidates

Results of the cost-benefit analyses were used to describe the relationship between profitability and number of selection candidates, length of the time horizon, and production output, and to estimate the optimum numbers of selection candidates. For the given index, breeding goal, and number of selected individuals, annual benefits were proportional to production output, genetic gain in TGC and TFC was proportional to $i$, and annual costs were assumed to be proportional to the number of selection candidates ($sc$). The accuracy of selection increases when the number of full- and half-sib records increases, but this effect was ignored because it was of minor importance: doubling the number of full- and half-sib records increased the accuracy by less than 2%. Genetic gain in M was independent of the number of selection candidates, because it was determined by the surviving proportion (“Appendix 2”). Rewriting Eq. 17 as a function of production output $P$ (tons/year), $i$, and $sc$, gives:

18 $$NPV_{appr} \left( {t, P, i, sc} \right) = \frac{P}{5000} \cdot \left( {i \cdot \bar{B}_{{{\text{TGC}}, {\text{TFC}}}}^{{\prime }} + \bar{B}_{\text{M}} } \right) \cdot \left( {\frac{{r \cdot \left( {r^{y} - r^{t} } \right)}}{{\left( {1 - r} \right)^{2} }} - \frac{{\left( {t - y} \right) \cdot r^{t + 1} }}{1 - r}} \right) - sc \cdot C^{{\prime }} \cdot \frac{{r - r^{t + 1} }}{1 - r},$$

where $\bar{B}_{{{\text{TGC}}, {\text{TFC}}}}^{{\prime }}$ is the average increase in annual benefits from genetic gain in TGC and TFC per unit of selection intensity $i$, $\bar{B}_{\text{M}}$ is the average increase in annual benefits from genetic gain in M, and $C'$ are average annual costs per surviving selection candidate. From $sc$, $i$ can be approximated algebraically as [23]:

19 $$i = 0.80 + 0.41 \cdot { \ln }\left( {sc/sel - 1} \right),$$

where $sel$ is 80 selected animals for breeding programs with multiplier tier and 175 for the breeding program without a multiplier tier. For simplicity, effects of $i$ on genetic variation were ignored. $NPV_{appr} \left( {t, P, i,sc} \right)$ was calculated for a time horizon of 10 years, a production output of 5000 tons, and a range of selection candidates.

Investment was optimum when $\delta NPV_{appr} \left( {t, P, i,sc} \right)/\delta sc = 0$ [5]. From Eqs. 18 and 19 and $\delta NPV_{appr} \left( {t, P, i,sc} \right)/\delta sc = 0$ at optimum investment, optimum $sc$ ($sc_{opt}$) was calculated based on [5] as:

20 $$sc_{opt} = \frac{P}{5000} \cdot 0.41 \cdot \bar{B}_{{{\text{TGC}}, {\text{TFC}}}}^{{\prime }} \cdot {{\left( {\frac{{r \cdot \left( {r^{y} - r^{t} } \right)}}{{\left( {1 - r} \right)^{2} }} - \frac{{\left( {t - y} \right) \cdot r^{t + 1} }}{1 - r}} \right)} \mathord{\left/ {\vphantom {{\left( {\frac{{r \cdot \left( {r^{y} - r^{t} } \right)}}{{\left( {1 - r} \right)^{2} }} - \frac{{\left( {t - y} \right) \cdot r^{t + 1} }}{1 - r}} \right)} {\left( {C^{{\prime }} \cdot \frac{{r - r^{t + 1} }}{1 - r}} \right)}}} \right. \kern-0pt} {\left( {C^{{\prime }} \cdot \frac{{r - r^{t + 1} }}{1 - r}} \right)}} + sel.$$

The number of selection candidates was optimized for a production output of 5000 tons per year at varying time horizon lengths, and for varying production outputs at a time horizon of 10 years. Using these estimated optimum numbers of selection candidates, $NPV_{appr} \left( {t, P, i,sc} \right)$ was estimated from Eq. 18, where $i$ was approximated from Eq. 19.

## Results

### Genetic level over time

For the nucleus in the baseline breeding program, genetic gain for each trait due to a single selection round is in Table 2. The selection differential ($S$) was €198/ton production, of which €180/ton was due to improvements in TGC and TFC ($S_{{{\text{TGC}},{\text{TFC}}}}$) and €18/ton was due to improvement in M ($S_{\text{M}}$). Simulations in the bio-economic model resulted in a selection differential of €197/ton production, indicating that non-linearity and trait interactions were negligible. For comparison, the product price received by the fish farming company was €4500/ton.

**Table 2:** Genetic gain per trait due to a single selection round in the nucleus of the baseline breeding program

Trait	Genetic gain (trait units)	Genetic gain (€/ton production)
TGC	1.089	436
TFC	0.568	− 256
M	− 0.0023	18
Total		198

TGC = Thermal growth coefficient (g^2/3^/(day degrees·1000))

TFC = Thermal feed intake coefficient (g^0.544^/(day degrees·1000))

M = Mortality rate (%/day)

**Fig. 2:**
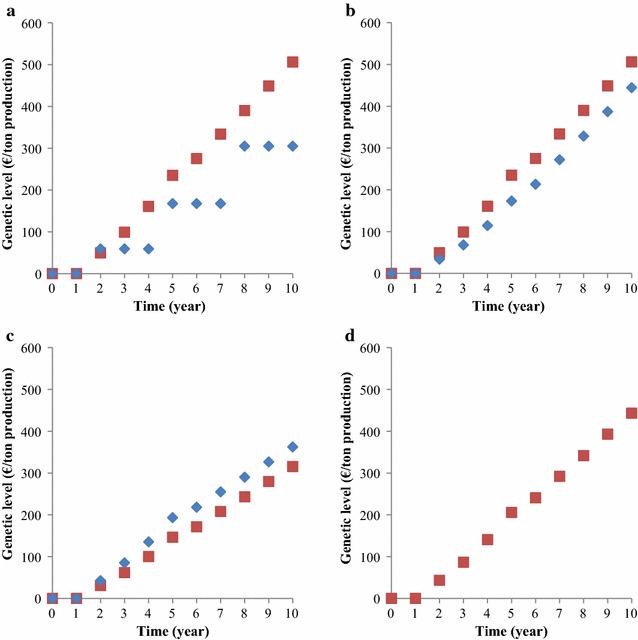
Increase in the genetic level in the nucleus (red square) and multiplier tier (blue diamond) for the four breeding programs over time. **a** Baseline breeding program, **b** breeding program with annual multiplier tier replacement, **c** breeding program with priority on improvement of the multiplier tier, **d** breeding program without a multiplier tier

Mean genetic levels of the nucleus and multiplier tier over 10 years from the start of breeding programs are in Fig. 2. In the baseline breeding program, the mean genetic level of the nucleus increased at a more or less constant rate, while the mean genetic level of the multiplier tier increased stepwise once every three years. For longer time horizons, the increase in mean genetic levels approached €56.5/ton production per year in the nucleus and €169/ton production per three years in the multiplier tier.

**Fig. 3:**
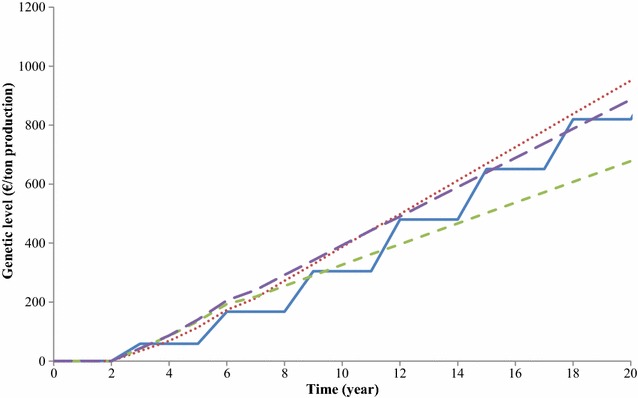
Increase in the genetic level of animals used in production for the four breeding programs over time. Blue line: baseline breeding program, red dotted line: breeding program with annual multiplier tier replacement, green dashed line: breeding program with priority on improvement of the multiplier tier, violet dashed line: breeding program without a multiplier tier

Genetic levels of animals used in production over 20 years from the start of breeding programs are in Fig. 3. The genetic level of animals used in production was highest up to year 10 in the breeding program without a multiplier tier. Thereafter, the genetic level of the breeding program with annual multiplier tier replacement was highest. The genetic level of animals used in production was higher up to year 7 for the breeding program with priority on improvement of the multiplier tier than for both the baseline breeding program and the breeding program with annual multiplier tier replacement (with nucleus priority).

### Cost-benefit analyses

Annual costs of the breeding programs are in Table 3, totalling €149,556/year for the breeding programs with a multiplier tier and €127,845/year for the breeding program without a multiplier tier.

**Table 3:** Annual costs of breeding programs

Item	With a multiplier tier (€/year)	Without a multiplier tier (€/year)
Buildings and tanks	9844	3411
Energy, oxygen, fuel	16,658	16,658
Husbandry nucleus	23,442	10,000
Rearing selection candidates	10,916	9081
Trait recording	4196	4196
External services	84,500	84,500
Total	149,556	127,845

**Fig. 4:**
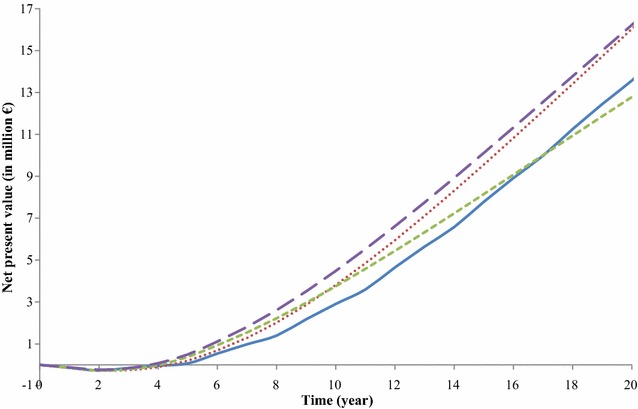
Development of the net present value of the four breeding programs over time. Blue line: baseline breeding program, red dotted line: breeding program with annual multiplier tier replacement, green dashed line: breeding program with priority on improvement of the multiplier tier, violet dashed line: breeding program without a multiplier tier

For the baseline breeding program, $NPV$ became positive after five years, reached 2.9 million € in year 10, and 13.6 million € in year 20 (Fig. 4). The baseline breeding program had the lowest $NPV$ up to year 17. After year 17, the breeding program with priority on improvement of the multiplier tier had the lowest $NPV$. The breeding program without a multiplier tier had the highest $NPV$ up to year 22. After year 22, the breeding program with annual multiplier tier replacement had the highest $NPV$.

For the baseline breeding program, the average increase in annual benefits ($\bar{B}$) over time approached 56.5·5000 = €282,000 per year. The average delay ($y$) between costs made for a selection round and resulting benefits was 4.2 years. $NPV_{appr} \left( t \right)$ could thus be calculated from Eq. 17 as:

21 $$NPV_{appr} \left( t \right) = 282,000 \cdot \left( {\frac{{0.94 \cdot \left( {0.76 - 0.94^{t} } \right)}}{0.0037} - \frac{{\left( {t - 4.2} \right) \cdot 0.94^{t + 1} }}{0.061}} \right) - 149,556 \cdot \frac{{0.94 - 0.94^{t + 1} }}{0.061} .$$

For the breeding program with annual multiplier tier replacement, $y$ was 2.5 years and $\bar{B}$ was €282,000 per year. For the breeding program with priority on improvement of the multiplier tier, $y$ was 0.4 years and $\bar{B}$ was €176,000 per year. For the breeding program without a multiplier tier, $y$ was 2.1 years and $\bar{B}$ was €247,000 per year.

### Optimum number of selection candidates

**Fig. 5:**
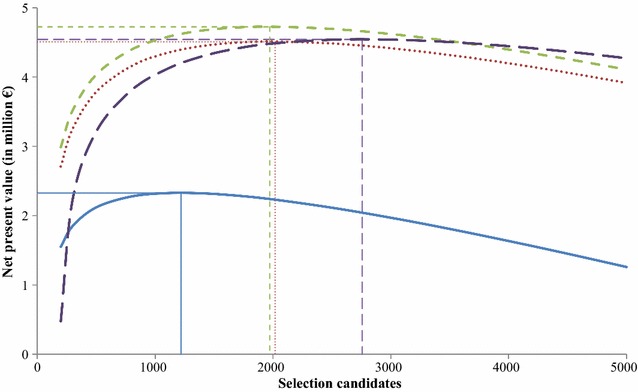
Net present value for the four breeding programs as a function of the number of selection candidates for a time horizon of 10 years and production of 5000 tons/year. Horizontal and vertical line segments indicate optima. Blue line: baseline breeding program, red dotted line: breeding program with annual multiplier tier replacement, green dashed line: breeding program with priority on improvement of the multiplier tier, violet dashed line: breeding program without a multiplier tier

For a 10-year time horizon and a production output of 5000 tons, $NPV_{appr} \left( {t, P, i, sc} \right)$ increased with the number of selection candidates up to an optimum, after which it gradually decreased (Fig. 5). Optimum numbers of selection candidates were 1218 in the baseline breeding program, 2015 in the breeding program with annual multiplier tier replacement, 1970 in the breeding program with priority on improvement of the multiplier tier, and 2756 in the breeding program without a multiplier tier. Any deviation from the optimum number of selection candidates was at the expense of $NPV$, although using too many selection candidates relative to the optimum led to a lower reduction of $NPV$ than using too few selection candidates.

**Fig. 6:**
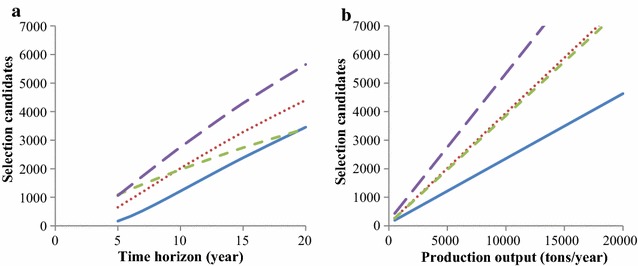
Optimum number of selection candidates for the four breeding programs. **a** As function of length of the time horizon for a production of 5000 tons/year, **b** as function of production output for a time horizon of 10 years. Blue line: baseline breeding program, red dotted line: breeding program with annual multiplier tier replacement, green dashed line: breeding program with priority on improvement of the multiplier tier, violet dashed line: breeding program without a multiplier tier

The optimum number of selection candidates increased with time horizon and production output, as presented in Fig. 6. Optimum numbers differed between breeding programs. The 2125 selection candidates in the baseline breeding program was optimum for a 14-year time horizon.

**Fig. 7:**
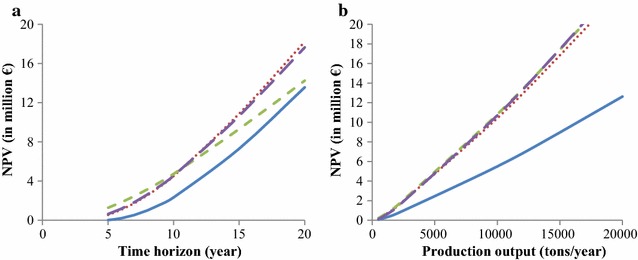
Net present value ($NPV$) for the optimum number of selection candidates in the four breeding programs. **a** As function of length of the time horizon for a production of 5000 tons/year, **b** as function of production output for a time horizon of 10 years. Blue line: baseline breeding program, red dotted line: breeding program with annual multiplier tier replacement, green dashed line: breeding program with priority on improvement of the multiplier tier, violet dashed line: breeding program without a multiplier tier

The $NPV$ per breeding program for optimum numbers of selection candidates are in Fig. 7. With optimum numbers of selection candidates and a production output of 5000 tons (Fig. 7a), the breeding program with priority on improvement of the multiplier tier had the highest $NPV$ up to year 10. Thereafter, the breeding programs with annual multiplier tier replacement and without a multiplier tier were superior. With optimum numbers of selection candidates and a time horizon of 10 years (Fig. 7b), $NPV$ was similar across breeding programs for any production output, except for the baseline breeding program, which had a much lower $NPV$.

## Discussion

### Cost-benefit analyses

In the baseline breeding program, part of the multiplier tier was replaced only once every three years. Annual multiplier tier replacement results in a consistently higher $NPV$, because the average delay between costs made for a selection round and resulting benefits is shorter. In the short term, putting priority on improvement of the multiplier tier over the nucleus resulted in a relatively fast increase in the average breeding value of animals used in production. This explained the higher $NPV$ of the breeding program with priority on improvement of the multiplier tier for the first 9 years relative to the breeding program with annual multiplier tier replacement (with nucleus priority). In the long term, however, the genetic level of animals used in production is determined by the genetic selection differential in the nucleus and putting priority on improvement of the multiplier tier is at the expense of the genetic selection differential in the nucleus. This explains the lower $NPV$ in the long term of the breeding program with priority on improvement of the multiplier tier relative to the breeding program with annual multiplier tier replacement (with nucleus priority).

A multiplier tier causes substantial delay in benefits from genetic improvement. Compared to the baseline breeding program and the breeding program with annual multiplier tier replacement, the breeding program without a multiplier tier had a lower genetic selection differential but the average delay between costs made for a selection round and resulting benefits was shorter. The shorter delay more than compensated for the reduction in genetic selection differential, resulting in a higher $NPV$ for the first 22 years. This has important implications for the design of breeding programs. In livestock species, a multiplier tier is used to disseminate genetic progress, to exploit heterosis, and to create market-specific crosses. In aquaculture breeding programs, selection in multiple pure lines is not common, except for carp [1], hence a multiplier tier does not offer any advantage with respect to heterosis or creation of specific line crosses. For most fish species, except salmonids, a multiplication tier is also not required to disseminate genetic progress because, generally, fish have a high fecundity rate [24]. Nevertheless, some integrated breeding companies use a multiplier tier, for example, to maintain a high level of biosecurity in the nucleus, and for flexibility in juvenile production. Such advantages should be weighed against the loss in $NPV$ of having a multiplier tier.

Results for the optimum number of selection candidates illustrate the importance of production output and time horizon as design parameters for breeding programs. A higher production output and a longer time horizon generate higher benefits, which, in turn, warrant higher annual costs. When the number of selection candidates was optimized, the breeding program with priority on improvement of the multiplier tier had the highest $NPV$ during the first 10 years but for time periods beyond this, other breeding programs were superior. These results demonstrate that re-ranking of breeding programs can occur with increasing time horizon. For the breeding programs considered, no re-ranking occurred with increasing production output, regardless of the time horizon.

Our results apply specifically to integrated breeding companies. Nevertheless, investments in a breeding program must be cost effective also for specialized breeding companies, because budgets are always limited and competitiveness of a breeding company is determined by the genetic level of its products. On the one hand, specialized breeding companies often supply a relatively large proportion of the market compared to integrated breeding companies [2], which warrants relatively large investments in the breeding program. On the other hand, specialized breeding companies accrue only a proportion of benefits obtained from genetic improvement. Thus, for a given production output, the optimum level of investment in a breeding program by a specialized breeding company may be lower than for an integrated company. Ultimately, investment in a breeding program by specialized breeding companies is driven by the expected outcome of the complex dynamics between the genetic level of its products relative to the competition, market shares, and the extent to which premium products fetch premium prices [25].

For aquaculture breeding programs, only a few cost-benefit analyses have been performed [26–28]. In all these studies, the profit equation that was used to derive economic values of traits did not provide an adequate description of the farming system [15]. Thus, the resulting economic values led to biased estimates of gains in farm profit after genetic improvement, and benefits could not be accurately estimated. Ponzoni et al. [26] and Ponzoni et al. [27] performed cost-benefit analyses of national breeding programs for Nile tilapia and common carp, respectively. Because benefits of these programs were distributed nationwide, they were highly profitable. When genetic improvement is disseminated nationwide, most benefits will accrue to consumers, because in an open and competitive market, lower production costs will be followed by lower market prices of fish. This is different from our study, where genetic improvement affects only production costs of a single company. Zuniga-Jara and Marin-Riffo [28] performed a cost-benefit analysis of a breeding program for abalone for an integrated company and their results were much less favourable than ours, largely because investments to expand production capacity were considered to be costs of the breeding program. The profitability of breeding programs in aquaculture has, in multiple occasions, been reviewed based on the (discounted) benefit-cost ratio e.g. [29–31]. Although this ratio is appealing, it may not be an appropriate evaluation criterion for breeding programs, because it favours underinvestment. For example, decreasing the optimum number of selection candidates by 10% results in a higher benefit-cost ratio, whereas it is at the expense of $NPV$ by definition.

### Genetic selection differential and rate of inbreeding

Economic values used in this study were derived for a situation in which production output of a farm is limited by stocking density [15]. Consequently, the genetic selection differential resulted from both a cost reduction per unit product and an increase in production output. Throughout Europe, different regulations apply that constrain production output of farms, and economic values depend on these quota [32]. If the quota were on production output instead of stocking density, improvement of TGC would not increase production output. Consequently, the economic value of TGC and the genetic selection differential would be lower than in the current study. We also assumed that the increased production output due to genetic improvement did not affect the market price of fish. This assumption is justified when the relative increase in supply is small and slow, and is absorbed by increasing demand [33], i.e. when the company produces only a small fraction of the total supply. Moreover, any price effect would be similar for all alternative breeding programs compared, i.e. would have only a limited impact on their relative performance. Thus, in our view, this assumption is justified.

Estimation of the genetic selection differential was subject to some simplifications. Equal contributions of year classes in the nucleus were assumed, whereas skewed contributions could be expected because average breeding values of older year classes were lower than those of younger year classes. The rate of genetic gain would benefit from a larger contribution of parents from younger year classes with higher average breeding values. For example, the extreme case with maximal contributions from age classes 2 (males) and 4 (females) of the nucleus, such that $$P_{1,j} = R_{1,j} = \left[ {\begin{array}{*{20}l} 0 \hfill & {0.5} \hfill & 0 \hfill & {0.5} \hfill & 0 \hfill \\ \end{array} } \right]$$ (top rows of Eqs. 2 and 6) resulted in a 2.6% higher $NPV$ in year 10. However, this would also result in an increase in the rate of inbreeding, but this could be controlled by balancing contributions of year classes. Thus, taking unequal contributions of year classes into account would make the prediction of the genetic selection differential unnecessarily complex.

The assumed genetic parameters imply that feed conversion ratio (FCR) improves as a correlated response to selection on TGC. However, there is some debate about the effect of selection on growth on FCR in fish, see review in [34]. Although a reduction in FCR is expected, it is useful to predict the genetic selection differential when there is no correlated response in FCR. For the predicted genetic gain in TGC of 1.089 g^2/3^/(day degrees·1000) (Table 2), genetic gain in TFC was increased in the bio-economic model up to the level that the resulting value of FCR was equal to FCR at levels of TGC and TFC before genetic improvement. The resulting value for genetic gain in TFC was 0.70 g^0.544^/(day degrees·1000), which was higher than genetic gain in the baseline breeding program. Thus, this value for genetic gain in TFC may be considered the maximum correlated response for the predicted genetic gain in TGC. If there is no correlated response in FCR, the selection differential in the baseline breeding program would be €137/ton production, i.e. a reduction of 31%.

For genetic gain in M, we assumed own performance selection on binomial records (dead or alive), such that all surviving selection candidates had equal estimated breeding values for M. Genetic gain would be higher when family information are used. With family information, all surviving selection candidates within a family would have equal estimated breeding values allowing for between family selection only. A possible correlated response in M from selection on TGC was ignored, leading to a conservative estimate of benefits due to improvement of M. Traits that are genetically correlated to survival could be used in the index for within-family selection. Several studies have reported positive genetic correlations between growth rate and survival, indicating that growth rate can be used as a selection criterion to improve survival [35–38]. These studies analysed only direct genetic effects and ignored indirect genetic effects on group mates, although these may explain a substantial part of the heritable variation [39]. A negative correlation of − 0.79 between the direct genetic effect of survival and indirect genetic effect of growth rate in a competitive environment suggests that an individual that has a high breeding value for survival will have a negative effect on the growth rate of its group mates [40]. Similarly, competition may also lead to a negative correlation between the direct genetic effect of growth rate and the indirect genetic effect of survival, but as far as we know, this correlation has not been studied in fish. A negative correlation between the direct genetic effect of growth and indirect genetic effect of survival would imply that faster growth in one fish may be at the expense of survival of its group mates. In case of competition, this correlation may be antagonistic to the correlation between the direct genetic effects of growth and survival. Thus, when indirect genetic effects are ignored, correlated response in survival when selecting on growth rate may be overestimated. Because we ignored a potential negative correlation between the direct genetic effect of growth and indirect genetic effect of survival, we also ignored the positive correlation between the direct genetic effects of growth and survival.

The breeding program of Andromeda S.A. is used to supply many different production sites across the Mediterranean. Thus, conditions for rearing selection candidates differ from those in production, which may cause genotype-by-environment interactions. Differences in the temperature regime at different sites can affect economic values of traits [41] and absolute changes in economic values directly affect the genetic selection differential. Both genotype-by-environment interaction and changes in relative economic values would lead to re-ranking of selection candidates, which would decrease the genetic selection differential. Furthermore, the genetic selection differential was assumed to be constant over generations. In reality, this is unlikely, because both genetic gain in individual traits and economic values may be non-linear over multiple generations. On the one hand, genetic gain in TGC and TFC was based on equilibrium genetic parameters, based on the Bulmer effect, hence the genetic selection differential that we used is conservative for the first generations. On the other hand, genetic gain in M is assumed to be constant, whereas constant genetic gain on the liability scale of cumulative mortality results in exponentially decreasing genetic gain on the observed scale for M. Thus, the genetic selection differential due to improvement of M would decrease over generations.

Each parental pair was assumed to have the same chance to produce offspring. In reality, the numbers of offspring per parent are skewed, which would increase the rate of inbreeding. Rate of inbreeding is managed by a mating design that controls parental contributions to replacement stock. Measures to control the rate of inbreeding may reduce the genetic selection differential. Compared to the baseline breeding program, all alternative breeding programs had an equal number of parents per selection round, an equal number of selection candidates, and an equal or lower selection intensity. Thus, each breeding program evaluated had similar or lower rates of inbreeding than the baseline breeding program.

## Conclusions

The baseline breeding program had a positive net present value after 5 years and was highly profitable thereafter. For a short time horizon, putting priority on improvement of the multiplier tier over the nucleus is more profitable than putting priority on nucleus improvement, and vice versa for a long time horizon. Use of a multiplier tier increases the delay between costs made for selection and resulting benefits. Thus, avoiding the use of a multiplier tier will increase the profitability of the breeding program in the short term. The number of selection candidates can be optimized to maximize net present value of a breeding program and this optimum increases with the length of the time horizon and production output. Using too many selection candidates relative to the optimum leads to less reduction in profitability than using too few selection candidates.

## Appendix 1: Genetic parameters of TGC and TFC

Coefficients and calculations are based on Janssen et al. [15]. For TGC, genetic variance ($\sigma_{{{\text{A}},{\text{TGC}}}}^{2}$) and phenotypic variance ($\sigma_{{{\text{P}},{\text{TGC}}}}^{2}$) were estimated from variation in harvest weight (${\text{HW}}$) and sum of effective temperatures at harvest, which was 4084 day degrees. For bodyweight, a genetic coefficient of variation of 10.6% and heritability of 0.34 were assumed [42]. Mean harvest weight ($\mu_{\text{HW}}$) was 400 g, hence genetic variance ($\sigma_{{{\text{A}},{\text{HW}}}}^{2}$) was 1798 g^2^ and phenotypic variance ($\sigma_{{{\text{P}},{\text{HW}}}}^{2}$) was 5288 g^2^. Genetic and phenotypic distributions of HW were simulated in R as ${\text{HW}}_{{{\text{A}},n}} = \mu_{\text{HW}} + z_{n} \cdot\sigma_{{{\text{A}},{\text{HW}}}}$ and ${\text{HW}}_{{{\text{P}},n}} = \mu_{\text{HW}} + z_{n} \cdot\sigma_{{{\text{P}},{\text{HW}}}}$, where subscripts ${\text{A}}$ and ${\text{P}}$ indicate genetic and phenotypic, and $z_{n}$ was a random number drawn from a standard normal distribution ($z_{n} \sim N\left( {0,1} \right)$) with $n$ = 1, …, 10^6^. From these distributions, $\sigma_{{{\text{A}},{\text{TGC}}}}^{2}$ and $\sigma_{{{\text{P}},{\text{TGC}}}}^{2}$ were estimated as:

22 $$\begin{aligned} \sigma_{{{\text{A}},{\text{TGC}}}}^{2} & \approx Var({\text{TGC}}_{{{\text{A}},n}} ) \approx \left( {\frac{1000}{4084}} \right)^{2} \cdot Var\left( {{\text{HW}}_{{{\text{A}},n}}^{2/3} } \right) \\ & = \left( {\frac{1000}{4084}} \right)^{2} \cdot \frac{1}{{10^{6} - 1}} \cdot \mathop \sum \limits_{i = 1}^{{10^{6} }} ({\text{HW}}_{{{\text{A}},i}}^{2/3} - 400^{2/3} )^{2} \\ & = 0.887({\text{g}}^{2/3} /({\text{day}}\,{\text{degrees}} \cdot 1000))^{2} , \\ \end{aligned}$$

and

23 $$\begin{aligned} \sigma_{{{\text{P}},{\text{TGC}}}}^{2} & \approx Var({\text{TGC}}_{{{\text{P}},n}} ) \approx \left( {\frac{1000}{4084}} \right)^{2} \cdot Var\left( {{\text{HW}}_{{{\text{P}},n}}^{2/3} } \right) \\ & = \left( {\frac{1000}{4084}} \right)^{2} \cdot \frac{1}{{10^{6} - 1}} \cdot \mathop \sum \limits_{i = 1}^{{10^{6} }} ({\text{HW}}_{{{\text{P}},i}}^{2/3} - 400^{2/3} )^{2} \\ & = 2.64({\text{g}}^{2/3} /({\text{day}}\,{\text{degrees}} \cdot 1000))^{2} . \\ \end{aligned}$$

For TFC, genetic variance ($\sigma_{{{\text{A}},{\text{TGC}}}}^{2}$) and phenotypic variance ($\sigma_{{{\text{P}},{\text{TGC}}}}^{2}$) were estimated from variation in stocking weight *(SW*) and variation in cumulative feed intake (CFI). Mean stocking weight ($\mu_{\text{SW}}$) was 4.4 g, hence genetic variance ($\sigma_{{{\text{A}},{\text{SW}}}}^{2}$) was 0.22 g^2^ and phenotypic variance ($\sigma_{{{\text{P}},{\text{SW}}}}^{2}$) was 0.64 g^2^. Genetic and phenotypic distributions of SW were simulated in *R* as ${\text{SW}}_{{{\text{A}},n}} = \mu_{\text{SW}} + z_{n} \cdot\sigma_{{{\text{A}},{\text{SW}}}}$ and ${\text{SW}}_{{{\text{P}},n}} = \mu_{\text{SW}} + z_{n} \cdot\sigma_{{{\text{P}},{\text{HW}}}}$, where subscripts ${\text{A}}$ and ${\text{P}}$ indicate genetic and phenotypic, and $z_{n}$ was a random number drawn from a standard normal distribution ($z_{n} \sim N\left( {0,1} \right)$) with $n$ = 1, …, 10^6^. Genetic and phenotypic correlations between SW and HW were assumed to be zero [43]. Between HW and CFI, a genetic correlation ($r_{\text{A}}$) of 0.9 and phenotypic correlation ($r_{\text{P}}$) of 0.8 were assumed [44, 45]. The regression of CFI on gain in bodyweight ($b_{{{\text{CFI}},{\text{HW}} - {\text{SW}}}}$) had a coefficient of 1.75 g feed/g gain and an intercept of 20 g feed. For CFI, genetic variance ($\sigma_{{{\text{A}},{\text{CFI}}}}^{2}$) and phenotypic variance ($\sigma_{{{\text{P}},{\text{CFI}}}}^{2}$) were calculated as:

24 $$\sigma_{{{\text{A}},{\text{CFI}}}}^{2} \approx \left( {\frac{{b_{{{\text{CFI}},{\text{HW}} - {\text{SW}}}} }}{{r_{\text{A}} }}} \right)^{2} \cdot \sigma_{{{\text{A}},{\text{HW}}}}^{2} = \left( {\frac{1.75}{0.9}} \right)^{2} \cdot 1798 = 6797{\text{g}}^{2} ,$$

and

25 $$\sigma_{{{\text{P}},{\text{CFI}}}}^{2} \approx \left( {\frac{{b_{{{\text{CFI}},{\text{HW}} - {\text{SW}}}} }}{{r_{\text{P}} }}} \right)^{2} \cdot \sigma_{{{\text{P}},{\text{HW}}}}^{2} = \left( {\frac{1.75}{0.8}} \right)^{2} \cdot 5288 = 25,301{\text{g}}^{2} .$$

Genetic and phenotypic distributions of CFI were simulated in *R* as:

26 $${\text{CFI}}_{{{\text{A}},n}} = 1.75 \cdot \left( {{\text{HW}}_{{{\text{A}},n}} - {\text{SW}}_{{{\text{A}},n}} } \right) + 20 + z_{n} \cdot\sigma_{{{\text{A}},{\text{CFI}}}} \cdot \sqrt {1 - r_{\text{A}}^{2} } ,$$

and

27 $${\text{CFI}}_{{{\text{P}},n}} = 1.75 \cdot ({\text{HW}}_{{{\text{P}},n}} - {\text{SW}}_{{{\text{P}},n}} ) + 20 + z_{n} \cdot\sigma_{{{\text{P}},{\text{CFI}}}} \cdot \sqrt {1 - r_{\text{P}}^{2} } ,$$

where $z_{n}$ was a random number drawn from a standard normal distribution ($z_{n} \sim N\left( {0,1} \right)$) with $n$ = 1, …, 10^6^. Negative values of ${\text{CFI}}_{{{\text{P}},n}}$ were removed ($n$ = 3). From these distributions, $\sigma_{{{\text{A}},{\text{TFC}}}}^{2}$ and $\sigma_{{{\text{P}},{\text{TFC}}}}^{2}$ were estimated as:

28 $$\begin{aligned} \sigma_{{{\text{A}},{\text{TFC}}}}^{2} & \approx Var({\text{TFC}}_{{{\text{A}},n}} ) \\ & = Var\left( {1000 \cdot \frac{{\left( {{\text{CFI}}_{{{\text{A}}, n}} + {\text{SW}}_{{{\text{A}},n}} } \right)^{0.544} - {\text{SW}}_{{{\text{A}},n}}^{0.544} }}{4084}} \right) \\ & = \left( {\frac{1000}{4084}} \right)^{2} \cdot \frac{1}{{10^{6} - 1}} \cdot \mathop \sum \limits_{i = 1}^{{10^{6} }} \left( {\left( {{\text{CFI}}_{{{\text{A}},i}} + {\text{SW}}_{{{\text{A}},i}} } \right)^{0.544} - {\text{SW}}_{{{\text{A}},i}}^{0.544} - \left( {\left( {713 + 4.4} \right)^{0.544} - 4.4^{0.544} } \right)} \right)^{2} \\ & = 0.304({\text{g}}^{0.544} /(1000\,^\circ {\text{C}}\,{\text{days}}))^{2} , \\ \end{aligned}$$

and

29 $$\begin{aligned} \sigma_{{{\text{P}},{\text{TFC}}}}^{2} & \approx Var({\text{TFC}}_{{{\text{P}},n}} ) \\ & = Var\left( {1000 \cdot \frac{{\left( {{\text{CFI}}_{{{\text{P}},n}} + {\text{SW}}_{{{\text{P}},n}} } \right)^{0.544} - {\text{SW}}_{{{\text{P}},n}}^{0.544} }}{4084}} \right) \\ & = \left( {\frac{1000}{4084}} \right)^{2} \cdot \frac{1}{{10^{6} - 4}} \cdot \mathop \sum \limits_{i = 1}^{{10^{6} - 3}} \left( {\left( {{\text{CFI}}_{{{\text{P}},i}} + {\text{SW}}_{{{\text{P}},i}} } \right)^{0.544} - {\text{SW}}_{{{\text{A}},i}}^{0.544} - \left( {\left( {713 + 4.4} \right)^{0.544} - 4.4^{0.544} } \right)} \right)^{2} \\ & = 1.17({\text{g}}^{0.544} /(1000\,^\circ {\text{C}}\,{\text{days}}))^{2} . \\ \end{aligned}$$

Heritabilities of TGC and TFC were calculated as $h^{2} = \frac{{\sigma_{\text{A}}^{2} }}{{\sigma_{\text{P}}^{2} }}$. Genetic and phenotypic correlations between TGC and TFC were confirmed to be equal to $r_{\text{A}}$ and $r_{\text{P}}$ between HW and CFI.

## Appendix 2: Selection response in M

On the underlying liability scale of cumulative mortality (CM), heritability ($h^{2}$) was assumed to be 0.17 [16], hence the genetic standard deviation ($\sigma_{\text{A}}$) was equal to $\sqrt {0.17}$. Before genetic improvement, the deviation of the threshold from the mean ($x_{B}$) was calculated from the quantile function of a normal distribution in *R* as:

30 $$x_{B} = qnorm\left( {0.149} \right) = - 1.04.$$

For mass selection on CM, $p = \frac{2125}{2500} = 0.85$, hence $i = - 0.27$. The deviation of the threshold from the mean after one generation of selection ($x_{A}$) was equal to:

31 $$x_{A} = x_{B} + i \cdot r_{{I{\text{H}}}} \cdot \sigma_{A} = - 1.04 - 0.27 \cdot \sqrt {0.17} \cdot \sqrt {0.17} = - 1.08.$$

On the observed scale, CM after selection was calculated from the distribution function of a normal distribution in *R* as:

32 $${\text{CM}} = pnorm\left( {x_{A} } \right) \cdot 100\% = pnorm\left( { - 1.08} \right) \cdot 100\% = 13.9\% ,$$

Hence M after one generation of selection was equal to:

33 $${\text{M}} = \left( {1 - \left( {1 - \frac{13.9}{100}} \right)^{{\frac{1}{540 - 1}}} } \right) \cdot 100 = 0.0278\,\% /{\text{day}}.$$

Thus, for the first selected generation, the response to selection on M was equal to $0.0278 - 0.0301 = - 0.0023$ %/day. For simplicity, the same response was assumed for successive generations.

## List of symbols

$B$: benefits in a given year (€)
$\bar{B}$: average increase in annual benefits (€/year)
$\bar{B}_{{{\text{TGC}}, {\text{TFC}}}}^{{\prime }}$: average increase in annual benefits from genetic gain in TGC and TFC per unit of $i$ (€/year)
$\bar{B}_{\text{M}}$: average increase in annual benefits from genetic gain in M (€/year)
$C^{{\prime }}$: average annual costs per surviving selection candidate (€/year)
$d$: discount rate (%/year)
$$i$$: selection intensity
M: mortality rate (%/day)
$NPV$: net present value (€)
$$NPV_{appr}$$: algebraically approximated $$NPV$$ (€)
$$p$$: selected proportion
*P*: production output (tons/year)
$$r$$: discount factor
$$sc$$: selection candidates
$sc_{opt}$: optimum number of selection candidates
$sel$: number of selected animals
$S$: genetic selection differential (€/selection round)
$S_{\text{M}}$: genetic selection differential due to improvement of M (€/selection round)
$S_{{{\text{TGC}},{\text{TFC}}}}$: genetic selection differential due to improvement of TGC and TFC (€/selection round)
$$t$$: time (year)
TGC: thermal growth coefficient (g^2/3^/(day degrees·1000))
TFC: thermal feed intake coefficient (g^0.544^/(day degrees·1000))
$y$: average delay between costs made for a selection round and resulting benefits (year)
